# Lookback supports semi-parallel, just-in-time processing in second language written composition

**DOI:** 10.1371/journal.pone.0334960

**Published:** 2025-11-03

**Authors:** Evgeny Chukharev, Jens Roeser, Mark Torrance

**Affiliations:** 1 Department of English, Iowa State University, Ames, Iowa, United States of America; 2 Department of Psychology, Nottingham Trent University, Nottingham, United Kingdom; National University of Malaysia Faculty of Education: Universiti Kebangsaan Malaysia Fakulti Pendidikan, MALAYSIA

## Abstract

**Purpose:**

Competent written production results from semi-parallel processes that flow from content determination to finger movement. Information from upstream processes that is not used immediately is easily lost. This disruption is more likely when a writer composes in a language in which they lack expertise (L2) than when they compose in their first language (L1). Looking back into text-just-written may serve to maintain fluent processing. Our research determined whether patterns of hesitation (pausing) and lookback in L1 and L2 writing were consistent with this theory.

**Method:**

39 university students composed argumentative texts by keyboard in L1 (English) and L2 (non-expert Spanish). Participants’ keystrokes were logged, and eye movements were tracked to determine when they looked back into their already-written text, and what text was fixated. Analysis focused just on cases where hesitation occurred in the absence of error correction.

**Results:**

Students hesitated more frequently and for longer when writing in L2. Lookback in L2 was more common and deeper within existing text before and within words. Lookback at start of sentences was relatively frequent in both L1 and L2, but more extensive in L2.

**Conclusion:**

Findings are consistent with lookback serving to refresh memory for what to write next.

## Introduction

Translating ideas into written language is in some ways more difficult and in some ways easier than speaking them. Writing is more difficult because it requires spelling retrieval and, typically, stricter adherence to formal grammatical conventions. Written communication is also asynchronous: Unlike conversational speech, in which speakers get immediate feedback on the communicational success of their utterances, writers must make their own decisions about the informational needs of their readers.

Writers have, however, two major advantages over speakers. First, they can pause when they want to – even mid-word – without compromising communication. Second, and related, writers can read what they have written, and therefore have rapid access to everything that they have already said. Writers take advantage of both of these affordances. Written production of extended text – composing an essay, for example – is marked by bursts of relatively fluent production followed by pauses [1]. During these pauses writers sometimes, though not always, look back into the text that they have already written [2–4].

The study that we report in this paper explored patterns in where and for how long writers pause during written composition, and the distribution of “lookback” events that sometimes accompany these pauses. Specifically, we examine differences in where and how often adult writers pause when composing in their first language and in a language that they are learning, in the extent to which these pauses are associated with lookback, and, when lookback occurs, in where writers look within their text. (Here and throughout we use the term *lookback*, in preference to *regression*, for any situation where the writer moves their eye away from the word that they are currently typing and looks back into the text that they have already produced. This may involve sustained reading but may also involve other patterns of eye movement. The operational definition of lookback used in the present study can be found in the Results section. *Written composition* refers to any situation where writer produces multi-sentence text in a context where they are responsible for content generation; contrast with, for example, copy typing.)

In presenting a rationale for our research we first discuss pause patterns in text production and provide a possible cognitive framework in which these can be interpreted. We then summarize the few existing studies that have explored lookback. Finally, we make a case for studying the ways in which lookback is affected by composing in a language in which the writer is not expert (henceforth *L2*).

### A semi-parallel process understanding of inter-keystroke intervals

Written output results from a set of processes that take the writer’s communicative intent and transforms it, via semantic, syntactic, lexical and orthographic processing, into motor plans for finger movements (e.g., [5]). As we have noted, the output rate fluctuates. In typewritten production this fluctuation can be captured by recording intervals between consecutive keystrokes. Explaining inter-keystroke intervals (IKI) requires a theory of how the various processes required to transform intent into keypresses are structured.

It may be that processing during writing is serial. Longer IKIs represent periods when the writer is planning what to write next, and what is written in the subsequent burst of fluent output is the result of this planning [6]. This account is consistent with the finding that average duration of IKIs immediately before sentence-initial keystrokes is longer than the average IKI before words, and that average IKI before words is, in turn, longer than the average IKI between mid-word key presses [7–9].

However although there is a greater tendency for longer sentence-initial IKIs these are still often very rapid. For example, Medimorec and Risko [10] found that undergraduate students writing on familiar topics had 71% of sentence-initial IKIs less than 1 s in duration. For comparison, this is less than mean response latency, in a similar population, for written naming of familiar objects [11]. Therefore, written composition is often remarkably fluent despite the fact that writers can pause when they want to, with impunity. This observation seems inconsistent with the assumption that pre-sentence pauses are associated with all planning necessary to determine content and structure of the following sentence.

Consistent with commonly held assumptions about spoken language production [12,13], several researchers have argued that the processes associated with written production run at least in part in parallel [5,14–16]. Van Galen, for example, argued for a sequence of modular processes, each responsible for a specific transformation (semantic, syntactic, and so forth), with processing at each level occurring as soon as information is input from the immediately upstream process [5] (see also [17]). Buffers provide transient storage at each processing level to accommodate unsynchronized output rates. Seeing composition as a semi-parallel process gives a different understanding of inter-keystroke intervals: IKIs result from one of two data-generating processes. When upstream processes output at a rate equal to or faster than can be used for finger-movement planning, then IKIs are determined just by time needed for executing finger movements (i.e., just by processing below the last buffer in the processing cascade). However, if one or more upstream processes provide output more slowly, then IKI becomes dependent directly on upstream processing times and not on time to move fingers. Therefore, IKIs form two distributions: one associated with rapid, fluent output, and another that results from delays caused by some combination of processing at the semantic, syntactic, lexical, or orthographic levels [18]. Roeser et al. [19], in an analysis of multiple datasets from writers composing multi-sentence texts, provide direct evidence for this two-process understanding of inter-keystroke intervals.

This semi-parallel processing account provides an explanation for why, as we noted above, writers sometimes pause before starting a sentence (or word), but often do not. The reason that writers sometimes do not hesitate at sentence boundaries is not because in some cases sentences are somehow produced with no advance planning but because planning was completed in parallel with previous output. Pauses therefore represent disfluency in the smooth flow of information through cognitive processes that lie upstream of finger-movement planning – cases where planning next texts spills beyond.

This disfluency is potentially disruptive. Writers can, as we have noted, pause at any point without this affecting the eventual communicative effect of their text. However, language processing is subject to what Christiansen and Chater describe as a fundamental “now-or-never” bottleneck [17]. Buffering is transient and as a consequence written (and spoken) production are just-in-time systems: Production must flow through a sequence of processes from message to finger movements without significant holdup. If there is delay – if, for example, a writer has to stop to mentally rearrange syntax or consciously retrieve a spelling – there is risk that output from upstream processes, particularly the chain of ideas that the writer wanted to communicate, will be lost. One possible function of lookback is to mitigate the consequences of this disruption.

### Lookback during written production

As we have noted, an essential difference between writing and speech is that writers can see what they have said. It is perhaps surprising, therefore, that writers’ eye movements within their already-written text has been the focus of only a small handful of studies [2–4,20,21]. This is partly for technical reasons. A text that is currently being composed is constantly changed, wrapped, and scrolled on the screen. This means that area-of-interest-based methods for locating fixations – a mainstay of reading research – cannot be used. Determining the target of a writer’s gaze – i.e., identifying the word and sentence that they are looking at within their own text at any particular point in time – requires triangulation of a fixation’s screen location with keystroke data to determine what text appeared at that location at the time that the fixation occurred. Solutions exist [2,3,22], but the number of published studies that use these methods is very small.

In possibly the most detailed description available of writers’ lookback behavior, Torrance and co-workers [4] found that, in adult writers composing argumentative essays, lookback into the already-produced text was relatively common. For example, a mean of 45% of sentence-initial keystrokes were preceded by the writers looking back into their existing text, reducing to 12% and 5% for word-initial and within-word keystrokes, respectively (Chukharev-Hudilainen et al. found similar frequencies for Turkish adult writers, see [2]). However, it was relatively rare for these lookback events to involve sustained reading. Under a weak definition of sustained reading that required a minimum of three consecutive forward saccades within the text, they found that sustained reading was associated with only 29% of occasions where writers looked back into their text before starting a sentence, 13% before starting a word, and 1% of the rare occasions where lookback occurred mid-word. A more typical pattern involved short sequences of fixations with gaze hopping back and forth between different words within the text. Although in some cases these fixations may have had no psycholinguistic significance – the words that are fixated may not have been processed – the researchers found that this was not generally the case: Gaze durations, even in “hopping” sequences, were predicted by the length of the fixated word.

Establishing where writers look back to within their texts may inform theory about lookback function. Deeper lookback – fixations at some distance from where the writer was most recently typing (the “point of inscription”) – may be associated with consideration of macrostructure and global coherence of the text. Where gaze is proximal to the last-typed character, it is more likely to be associated with locating errors or maintaining local coherence. Beers et al. found that lookbacks that focused on text within one line of the point of inscription were 1.6 times more likely than more distal lookbacks [21]. The location of the point of inscription (where the writer is at in their text when they look back) also seems important. Wengelin et al. found that mean distance, in words, of fixation location was considerably greater when looking back from sentence boundaries than from within or before a word [3]. They found that 80% of writers’ fixations were within 60 words of the point of inscription when this occurred at a sentence boundary, but within 22 words of the point of inscription when the last-typed character was before or within a word. Chukharev-Hudilainen et al. [2] found a similar effect.

Looking back into already-written text is likely to serve one or both of two broad functions. Most obviously, lookback serves to check or search for errors or, more generally, to review what has been written to make sure that it conforms to the writer’s communicational goals. As we have noted, however, the majority of lookback events are associated with hopping back and forth among isolated words rather than sustained sequences of forward saccades. These hopping events are arguably unlikely to function as error monitoring or review.

An alternative explanation is that lookbacks primarily support text planning by cueing retrieval of what to write next. As we have argued, a semi-parallel, just-in-time understanding of written production suggests that the writing process is easily disrupted. Buffers at each level of processing provide only transient storage. If output from upstream processes cannot be used promptly, then it is likely to be lost. Text-just-written necessarily provides a rich source of retrieval cues that can be exploited to permit rapid reinstatement of lost information: A writer who is about to start a mid-paragraph sentence but has, for example, been struggling with word choice in the previous sentence may find that they have forgotten what it was that they intended to communicate next. Glancing back at content words within the previous sentence is very likely to cue what they were going to say, allowing fluent processing to recommence.

### Lookback when writing in a second or other language

Writers’ eye movements when writing in a language in which they have not attained native-like proficiency (i.e., in L2) is interesting for two reasons. First, writing in L2 is a common activity for many people. Second, the need for not just error monitoring but also message reinstatement following output disruption will be greater when the writer does not have mastery of the language in which they are writing.

Several studies have used keyboard data to study production fluency in adults composing in L2 [9,23–29]. As might be expected, IKIs at different text locations follow a similar pattern to those found in the native language (L1), with durations increasing across within-word, word-initial, and sentence-initial locations [26,29]. There is some evidence that IKIs decrease with increasing language proficiency [25]. The few studies that have compared the same writers composing text in L1 and L2 have found some evidence of longer IKIs in L2 [30–32]. Van Waes and Leijten, for example, found a tendency towards longer within-word and word-initial IKIs, but no effect on sentence-initial IKIs [32].

To our knowledge, just three papers have explored lookback in writers composing text in L2. Gánem-Gutiérrez and Gilmore [33] captured eye movements of adult writers composing in L2 (English) and reported a mean of 15% of total time spent “rereading” (with eyes fixated somewhere within already-produced text). Révész et al. [28] recorded the eye movements of students composing short texts in L2 (English). All had Mandarin as L1 but sufficient competence in English to study at the master’s level or above at an English-speaking university. The authors identified IKIs of greater than 2 s and categorized them based on the location of the majority of fixations that occurred during the pause. Their findings suggest, broadly, that pauses between and within words were associated with similar eye movement patterns, with majority of fixations roughly equally likely to rest within the current sentence and within a previous sentence. Chukharev-Hudilainen et al. [2] studied eye movement of students at an English-speaking university in Türkiye who composed text in Turkish (L1) and English (L2). They found that the probability of lookback was greater in L2 than in L1.

### Present study

We studied writers composing texts under controlled conditions in English (L1, i.e., their first language) and Spanish (L2, i.e., a language in which they had not reached native-like competence). We captured the time of each keystroke and also the text-anchored location of each fixation when the writer’s gaze fell within the text that they had already written (i.e., the word, sentence, and paragraph that they were fixating). Our analysis differentiated lookback that was associated with making changes to previous text (i.e., error detection and correction), and lookback that was not accompanied by changes to existing text and therefore was at least plausibly associated with cuing what to write next. We focus on the latter.

We hypothesized effects of language status (L1/ L2) on IKIs and lookback as follows: Most obviously, we predicted more activity associated with error detection and correction in L2 than in L1. Specifically, in L2 we anticipated proportionally more keystrokes associated with making changes to existing text and a greater tendency for lookbacks into existing text to be followed by keystroke activity resulting in changes to existing text. However, following a cascading, just-in-time understanding of the organization of writing processes, we also anticipated a greater tendency for L2 writers to pause and to look back without then making changes to their text. Processes that are fluent and modularized in L1 (e.g., lexical and orthographic retrieval, grammatical inflection) are likely to be hesitant and require central processing in L2. L2 writing, particularly by writers with relatively low proficiency in L2, is also often accompanied by L1 inner speech [34]. L2 writing may also be prone to activation of lexemes in L1, particularly when L1 and L2 share many cognates [35,36], as was the case in the present study. For any or all of these reasons, we expected disruption to fluent production in L2.

We therefore predicted that writing in L2 would be accompanied by a greater number of longer IKIs resulting from a greater tendency for upstream processing to lag behind motor output. We also predicted a greater tendency for lookback in L2, without editing, as a strategy for recovering from this disruption.

A particular feature of this study was our use of Bayesian mixed-effects modeling and, for analysis of IKIs, Bayesian mixture models. Bayesian methods allowed us to make direct inferences about out-of-sample (population) effect size. Mixture models capture the assumption, discussed above, that the time between two specific keystrokes is best understood as belonging to one of two distinct distributions: (1) a distribution associated with fluent production, when the upstream processes are operating at sufficient speed for motor execution to be the limiting factor, and (2) a distribution representing longer IKIs where this flow is somehow interrupted [18]. This avoids the need both for somewhat arbitrary decisions about at what threshold an IKI becomes a “pause,” and for separate analysis of pause frequency and mean pause duration. Both are captured within a single model.

## Method

This study was approved by Iowa State University’s Institutional Review Board (IRB) and conforms to the U.S. Federal Policy for the Protection of Human Subjects. Recruitment for this study began on November 29, 2016, and was completed on December 9, 2016. All participants provided prior written informed consent. All participants were compensated for their participation in the study.

### Design and participants

Undergraduate university students (N = 39, mean age = 20.6 years, SD = 1.51) composed argumentative essays in English (L1) and Spanish (L2). Two students were completing Spanish courses at beginner level, 18 intermediate, and 17 advanced. Course data were missing for two students. All students had native-equivalent English language competence.

Students wrote within the CyWrite word-processing environment [2] which provides word processing functionality similar, for example, to Microsoft WordPad, including text selection by mouse action and copy-and-paste. CyWrite logs writers’ keystrokes and eye movements. Order and writing prompt (essay topic) were counterbalanced across languages.

### Writing tasks

Participants wrote, with a 40-minute time limit, to each of two prompts:

“Some people have said that music not only entertains us but changes how we think and feel about ourselves. Do you think music has the power to influence as well as to entertain people? Support your views with specific examples from your own experience, observations or reading. *Algunas personas dicen que la música no solamente nos divierte, pero también nos cambia el modo de pensar y sentir acerca de uno mismo.¿Usted piensa que la música tiene el poder de influir y de entretener a las personas? Dé su opinión con ejemplos de su experiencia, observaciones, y/o lecturas.*”

“Some people say that computer technology is a barrier to developing real friendships. To what extent do you agree or disagree with the statement above? Support your views with specific examples from your own experience, observations or reading. *Algunas personas dicen que la tecnología de las computadoras es una barrera para el desarrollo de amistades verdaderas.¿Hasta que punto está o no de acuerdo usted con la información de la oración anterior? Dé su opinión con ejemplos de su experiencia, observaciones, y/o lecturas.*”

Participants were asked to write five-paragraph essays, to a standard such that they would be happy to submit their essays for assessment.

### Equipment and procedure

We ran the CyWrite program on mid-range desktop PCs equipped with E2214hb Dell monitors with 1920 x 1080 pixel resolution and a visible screen size of 476 mm x 268 mm. CyWrite is written in JavaScript and runs within a web browser [2]. This permits online (remote) data collection. However, in the present study data were collected face-to-face and under laboratory conditions. Eye tracking was achieved using GazePoint GP3 devices, used without head restraint. This device has a 60 Hz sampling rate and observed spatial resolution of 1.25 vertical and 0.72 horizontal degrees of visual angle [37]. Participants sat approximately 580 mm from the eye tracker, with their horizontal gaze positioned at display center. The CyWrite editor window subtended a visual angle of 32.2 degrees horizontally and 18.5 degrees vertically. A five-letter word subtended a visual angle of 2.5 degrees, and line spacing was set at 2.0 degrees. Mean duration of fixations across all participants was 403 ms (SD = 367).

Participants completed both writing tasks in a single session, with a short break between tasks. They completed nine-point calibration prior to each task. All participants in our sample had average calibration errors of less than 1.8 degrees.

## Data and analysis

For each keystroke, including presses of delete and cursor keys, and for each mouse operation, the CyWrite program records the outcome of the action (e.g., adding a character to the existing text) and the precise time at which the action was initiated. The program also automatically maps eye movement data onto information about the current text state. This provides, for each fixation, the word that is being fixated and the location of that word within the emerging text as it was at the point in time when the fixation occurred.

Analysis of keystroke and eye data involved three steps. First, we identified different categories of text-output activity based on orthographic and temporal analysis of keystroke logs (broadly, what appeared on the screen, and when). These categories, which we will call *transition types*, were defined in terms of pairs of temporally consecutive events. (Previous studies that have explored text production time course, including our own, have used the term *text boundary* in this context. Here we use *transition* to capture the fact that in some cases the second event of the pair is associated with making changes to existing text rather the contributing to the front edge of the text currently under production.) Second, we then determined whether or not the writer looked back into their text in the period between the first and second event in the pair. So, for example, if a participant typed “b” then “a,” then fixated one or more words in their existing text, and then completed the word “barked,” that would constitute a mid-word transition with lookback. Third, just for transitions where lookback occurred, we calculated various measures of fixation location and duration that characterized the participant’s eye movement. Data processing was implemented in a combination of JavaScript and R code.

### Typing actions (Transition types)

It is possible to identify multiple different transition types. The particular subset explored in the present research follows analyses in previous studies (e.g., [2,4,8,38]) and is detailed in Table 1.

**Table 1:** Typing actions classification.

Transition type	Description
Within-word	*Preceding keypress*: Any letter. *Following keypress*: Any letter. *Example*: T^h^e d^o^g b^a^r^k^e^d. T^h^a^t[bsp][bsp]e^n i^t r^a^n a^w^a^y.
post-word^1^	*Preceding keypress*: Any letter. *Following keypress*: Space. *Example*: The^ dog^ barked. That[bsp][bsp]en^ it^ ran^ away.
pre-word	*Preceding keypress*: Space. *Following keypress*: Any letter. *Example*: The ^dog ^barked. That[bsp][bsp]en ^it ^ran ^away.
pre-sentence^2^	*Preceding keypress*: Space. *Following keypress*: Any letter. *Example*: The dog barked. ^That[bsp][bsp]en it ran away.
word→edit	*Preceding keypress*: Any letter, including word-terminal letters. *Following keypress*: Cursor move or deletion action. *Example*: The dog barked. That^[bsp][bsp]en it ran away.
pre-word→edit	*Preceding keypresses*: Any letter then space. *Following keypress*: Cursor move or deletion action. *Example*: The dug ^←←o→→ barked. Then it ran away.
pre-sentence→edit	*Preceding keypresses*: Sentence-terminating punctuation (e.g., period, question mark), then space. *Following keypress*: Cursor move or deletion action. *Example*: The dog barkid. ^←←←[bsp]e

Note. In examples ^ marks transition location, [bsp] represents backspace, ← and  → represent single-character backward and forward cursor moves, implemented by either mouse or cursor key. ^1^For reasons that we discuss, these were treated as within-word in our analyses. ^2^Producing uppercase letters requires two keypresses – shift and character. Time between shift and character keypresses was non-negligible. IKIs were timed to the shift keypress.

There are three points to note here. First, although the examples in Table 1 show very local editing, editing actions could occur at any distance from the character created by the preceding keystroke. A space press followed by a cursor move up several lines in the text, followed by insertion of a word would also be classified as a pre-word edit. Second, it is only possible to categorize editing transitions on the basis of the preceding keypresses. This means that whereas it is possible to differentiate between within-word and post-word non-editing transitions, whether a character that precedes an edit is within or at the end of a word is indeterminate. For this reason, we combined post-word and within-word categories, labeling all as “within-word.” Third, there were some cases where the writer moved the cursor, then returned it to exactly its previous location, and then continued typing. This action was particularly associated with the writer looking back into a long document with cursor movement used as a scrolling strategy. Cases where no editing occurred and cursor movement could be interpreted as supporting lookback were classified as the appropriate non-editing transitions.

As a measure of production fluency, we recorded production sequence length, where a production sequence was defined as a sequence of keystrokes that terminated with an editing action. Longer mean production sequence length indicates more fluent production.

### Eye movement

Our analysis of eye movement involved first identifying sequences of fixations that could be classified as lookback events. A lookback event comprised a sequence of two or more fixations that occurred between the first and second keyboard events in a transition where (a) fixations were located at a median distance of at least one word and at least 5 characters behind the location of the last-typed character (i.e., were not focused on the characters currently being typed), (b) more than 80% of fixations in the sequence were within text that preceded (rather than followed) the current location, (c) the lookback sequence did not start with a fixation on the text of the writing prompt (the text at the top of the screen describing the writing task), and (d) included fewer than 50% of fixations on the prompt.

### Statistical analysis

Data were modeled with Bayesian mixed-effects models using the R package *brms* [39] and the probabilistic programming language *Stan* [40]. All models were fitted with random intercepts for participants and with by-participant slope adjustments for the effect of Language (L1 vs. L2), and with weakly-informative priors. We report parameter estimates from these models with accompanying 95% probability intervals (95% PI).

We provide support for the presence of effects of interest with Bayes Factors (BF) calculated using the Savage-Dickey method [41,42]. BFs in our analyses represent the ratio of evidence in favor of a non-zero effect to evidence that the effect is zero. For example, BF = 2 indicates that the alternative hypothesis is twice as likely as the null hypothesis. By convention, BF > 5 indicates moderate evidence and BF > 10 indicates strong evidence in favor of a non-zero effect [43,44].

Transition durations were analyzed as finite mixture models, as described in Roeser et al. [18] (see also [45,46]), for reasons discussed in our introduction. These models adopted the same random-effect structure as other models, with the addition of by-participant mixing proportions. This captured variation across participants in the extent to which they are likely to pause when typing. Readers unfamiliar with these models can find equations in the appendix.

## Results

We detail our findings in three sections. First, we provide a brief overview of the characteristics of students’ completed texts. We then examine the frequency of writing and editing keystrokes by which these texts were created. These analyses provide necessary context for interpreting temporal patterns in participants’ keystrokes and lookbacks. These are then analyzed focusing just on activity during writing transitions (i.e., when the transition did not terminate with an editing action). We first model IKI duration and then patterns of lookback, in each case analyzing in terms of text location (before sentence, before word, within word).

### Text characteristics

As might be expected, and can be seen in Table 2, participants’ L2 texts were shorter, were composed of shorter sentences, were informationally more sparse (had lower ratio of open- to closed-class words), and were lexically less diverse (measured using the MTLD statistic [47]) as a sensitive and text-length independent measure of lexical diversity [48].

**Table 2:** Characteristics of texts written in L1 and L2. Mean (SD) and standardized effect of language (with 95% PI).

Measure	L1	L2	Language effect	BF
Word count	668 (301)	326 (154)	−1.17 [−1.54, −0.81]	>100
Mean word length	4.4 (0.20)	4.8 (0.30)	1.28 [0.92, 1.64]	>100
Sentence count	33.5 (15.9)	22.9 (9.9)	−0.75 [−1.17, −0.34]	94.41
Mean sentence length	20.7 (4.1)	14.2 (3.0)	−1.33 [−1.67, −0.99]	>100
Ratio open class to closed class words	1.61 (0.13)	0.91 (0.10)	−1.89 [−2.04, −1.74]	>100
Lexical diversity (MTLD)	81.3 (16.1)	59.0 (16.7)	−1.12 [−1.50, −0.75]	>100

Note. Parameter estimates and BFs from a multivariate linear model with language (L1 vs. L2) as predictor. Language effect is log scaled.

### Keystroke frequencies

Table 3 gives mean count keystrokes, by language, and by whether the next keystroke was ongoing production, or was an editing action (deletion or moving the cursor).

**Table 3:** Observed by-participant mean (SD) count of keystrokes, by language, by location in the text, and by whether the next keystroke was a continuation of the text (Writing) or the start of an action that revised existing text (Editing).

	Writing		Editing		Proportion Editing^1^	
	L1	L2	L1	L2	L1	L2
Before sentence	29 (14)	19 (9)	6 (5)	6 (4)	.15 [.12, .17]	.24 [.21, .27]
Before word	676 (283)	320 (154)	56 (35)	48 (30)	.07 [.06, .08]	.13 [.12, .14]
Within word	3,403 (1,385)	1,800 (793)	143 (75)	112 (56)	.05 [.04, .06]	.07 [.07, .08]

Note: ^1^Model estimates for proportion of keystrokes that initiated editing, with 95% PI.

It would seem probable that writing in L2 affects the likelihood that a writer will stop and edit their text, and that this is moderated by location within the text. To explore this possibility, we fitted a binomial model predicting proportion of editing transitions, with fixed effects for language and location. Location was modeled as two comparisons: before sentence vs. before word, and within word vs. before word or sentence. Estimates from this model can be found in the final two columns of Table 3. IMMoAs might be expected, there was a main effect of language, with a higher proportion of edits in L2 than in L1. For both languages, editing was more likely before sentence than before word, and least likely within words (BFs > 100). Language moderated the location effect, with substantially more editing in L2 than L1 at sentence and word boundaries, but less of an effect within word (BF = 40). Model parameter estimates can be found in S1 Appendix Table 1.

### Inter-keystroke intervals and lookback characteristics

Before analysis we removed transition durations ≤ 50 ms, which typically occur as a result of accidentally pressing two keys at once (*M* = 2.12%, of observations, averaged across participants and tasks *SE* = 0.29), and ≥ 30 s (very long hesitations typically associated with extended reading or off-task activity; *M* = 0.05%, *SE* = 0.01). For computational reasons, where number of observations per participant in a cell of this design exceeded 200, we reduced it to 200 by random sampling. Overall, this meant that our sample comprised IKIs at all before-sentence transitions, a mean of 50.48% of before-word transitions (*SE* = 8.01%), and a mean of 17.02% of within-word transitions (*SE* = 6.02%). Our focus in all remaining analyses is on behavior associated just with Writing transitions. Data for Editing transitions were not included in our models. (It is trivially true that Editing transitions will, in nearly all cases, involve writers looking back in their existing text. Eye activity is likely to involve either searching for or tracking the cursor back to the location of the error.)

#### Inter-keystroke intervals (transition durations).

As previously discussed, we modeled transition durations (time between first and second keypress) as finite mixture models ([49], pp. 519–543; [18,19]) with fixed effects for language and for location in text. Consistent with a cascading account of written production, we assumed two data-generating processes: one associated with finger movement (specifically any components of the writing process after the final buffer), and one for the case in which higher processes upstream lag behind finger-movement processing resulting in longer IKIs. Our model was parameterized so that the distribution of the short-duration IKIs (associated with finger movement) was constrained so as not to vary across language or text location, but was allowed to vary among participants, capturing the idea that individuals vary in their typing motor skill. Both the distribution of the longer mixture component and the mixing proportion were allowed to vary across all fixed and random factors. The distributional formula for this model can be found in the appendix.

**Fig 1:**
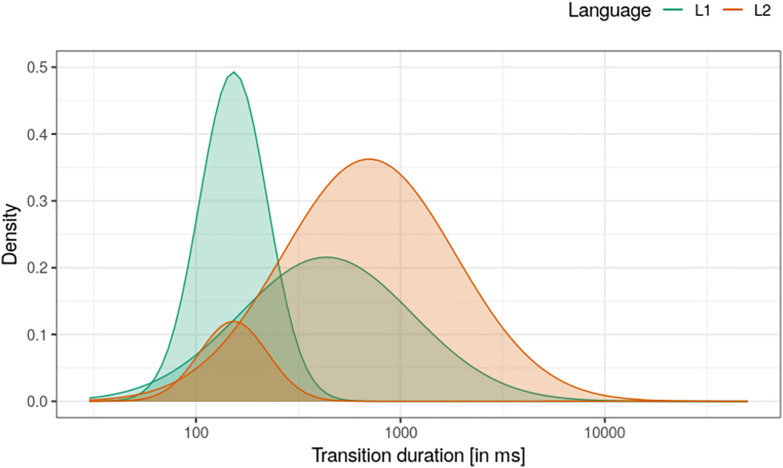
Estimated transition durations (inter-key intervals) before words, for participants writing in L1 and in L2. Data just for continuous-writing transitions (transitions that did not end in revision).

Fig 1 illustrates our approach with findings for just the before-word text location. This shows the following: in L1, most transitions were short, with relatively few longer durations (34% of total word-initial transitions). In L2, longer transitions were both more common (64%) and longer (estimated mean duration = 946 ms, compared to 536 ms for L1; see second row in Table 4).

**Table 4:** Estimated duration and proportion of longer IKIs (hesitations) at different text locations when participants wrote in L1 and in L2, with 95% PIs.

	L1	L2	Language effect	BF
Mean duration, ms				
before sentence	1,366 [1,199, 1,549]	1,912 [1,669, 2,180]	−0.34 [−0.49, −0.18]	> 100
before word	433 [400, 468]	703 [652, 756]	−0.48 [−0.54, −0.43]	> 100
within word	271 [204, 379]	370 [333, 413]	−0.32 [−0.58, −0.02]	1.09
Proportion				
before sentence	.78 [.70, .85]	.94 [.90, .97]	−1.44 [−2.19, −0.73]	> 100
before word	.53 [.43, .62]	.88 [.84, .92]	−1.95 [−2.53, −1.38]	> 100
within word	.08 [.04, .14]	.20 [.14, .27]	−1.07 [−1.79, −0.36]	32.6

Note. Data from just Writing transitions. Language effect is logit scaled for proportions and log scaled for durations. Estimates from a finite (two-component) mixture model. Short transition durations were constrained to be equal in both languages and at all locations. Estimated short-duration mean was 153 ms, 95% PI [142, 163].

Estimated cell means and mixing proportions for all text locations can be found in Table 4 (model parameter estimates are given in S1 Appendix Table 1). Hesitations (long transition durations) were both rare and relatively short within words, longer and more common before words, and very common and even longer at sentence boundaries (BFs > 100 for effect of text location on both long-interval duration, and mixing proportion). Hesitation was more common in L2 than in L1 at all three text locations. This was particularly the case before words, with 88% of words being preceded by a hesitation in L2 compared to only 53% in L1. Hesitations in L2 were also substantially longer than in L1 before sentences and before words, but with negligible evidence of an effect within words.

### Lookback probability

We first modeled the proportion of transitions in which participants looked back into their text between the first and second keypress, following the definition of lookback that we gave above. We fitted a binomial model with fixed effects for language and location. As in previous models, location was modeled as two comparisons: before sentence vs. before word, and within word vs. before word or sentence. Model parameter estimates can be found in S1 Appendix Table 3 and estimated cell means in Table 5.

**Table 5:** Lookback characteristics in L1 and L2 tasks during transitions at specific text locations.

	L1	L2	Language effect	BF
Proportion of transitions that included lookback				
Before sentence	.342 [.289, .408]	.396 [.32, .492]	−0.24 [−0.54, 0.06]	0.52
Before word	.041 [.033, .052]	.108 [.083, .149]	−1.05 [−1.29, −0.82]	> 100
Within word	.003 [.002, .004]	.008 [.006, .012]	−1.1 [−1.38, −0.84]	> 100
Mean lookback duration				
Before sentence	1,932 [1,677, 2,216]	2,711 [2,309, 3,162]	−0.34 [−0.52, −0.15]	49.4
Before word	1,153 [1,037, 1,274]	1,423 [1,281, 1,575]	−0.21 [−0.32, −0.11]	51.6
Within word	795 [707, 891]	1,004 [898, 1,119]	−0.23 [−0.36, −0.11]	31.7
Mean number of words fixated				
Before sentence	8.67 [7.82, 9.6]	11.26 [9.95, 12.73]	−0.29 [−0.45, −0.14]	> 100
Before word	4.42 [4.07, 4.78]	4.59 [4.18, 5.05]	−0.05 [−0.16, 0.06]	0.15
Within word	2.55 [2.24, 2.88]	2.6 [2.31, 2.94]	−0.06 [−0.24, 0.11]	0.23
Mean distance of most-fixated sentence from point of inscription^1^				
Before sentence	1.45 [1.29, 1.63]	1.68 [1.38, 1.99]	−0.14 [−0.33, 0.06]	0.55
Before word	0.54 [0.43, 0.67]	0.8 [0.64, 0.99]	−0.41 [−0.63, −0.2]	> 100
Within word	0.46 [0.34, 0.61]	0.85 [0.66, 1.07]	−0.65 [−0.96, −0.35]	> 100

Note. For Writing transitions only. Model estimates, with 95% PIs in parenthesis. Language effect is logit scaled for proportions and log scaled for other measures. ^1^Same sentence = 0, previous sentence = 1 etc. Note that minimum distance when transition is before sentence is 1, and therefore separate models for before-sentence, and for before and within word.

There was a clear and large effect of location (BFs > 100), with lookback over 10 times more likely before words than within words, although lookback before words was still relatively rare. Lookback before starting a sentence was much more common, occurring at around 1 in 3 sentence boundaries. Lookback both within words and before words was much more likely when participants wrote in L2. However, we found no evidence of a language effect on proportion of lookbacks at the start of sentences (BF > 100 for the interactions between location – before sentence vs. before word – and language).

#### Lookback duration.

We measured lookback duration as the total time that participants spent looking back into their text at a particular transition, for just those transitions where lookback occurred, calculated as the sum of the durations of all fixations during the lookback. We modeled this as a (unimodal) linear mixed effects model with, as in previous models, random intercepts for participants and by-participant slope adjustments for language (L1 vs. L2). Estimated cell means can be found in Table 5 and model parameter estimates can be found in S1 Appendix Table 4.

There was a general tendency for longer lookback durations for transitions at higher-level text boundaries. This effect was greater in L2 compared to L1 (BFs > 100 for the language by location interaction). At all three locations lookback duration tended to be longer in L2 relative to L1 (Table 5).

#### Number of words fixated.

Total number of words fixated is the sum of the times that the eyes moved to and fixated a different word during a lookback sequence, including cases where gaze exited a word but then returned to the same word at a later point in the sequence. This was modeled as a negative binomial with fixed and random effects as in previous models. Estimated cell means can be found in Table 5 and model parameter estimates in S1 Appendix Table 3.

We found strong evidence that number of words fixated was affected by the location of the transition, with more words fixated when the transition was before-sentence, then when before word, and fewest when the transition was within word (BFs > 100). There was no evidence of a difference in number of words fixated when lookback occurred during within-word or before-word transitions. However, there was a clear tendency for more extensive lookback at sentence boundaries when participants wrote in L2 (BF > 100).

### Depth of lookback

We explored how far back into their texts students looked, measured in number of sentences boundaries from the point of inscription (i.e., from the place in the text were the transition occurred). We first identified the sentence that, during the lookback sequence, received the most fixations, ignoring sequences where two or more sentences received the same number of fixations (i.e., where there was not a single modal value). For transitions that occurred at sentence boundaries – i.e., after a sentence had been terminated – then necessarily all fixations were at least in the previous sentence (a distance of 1 sentence), whereas for transitions before or within words it was possible for the most-fixated sentence to be the current sentence (a distance of 0 sentences). For this reason, we modeled before-sentence separately from before- and within-word transitions. We again modeled as a negative binomial, with main and fixed effects as in previous models. Parameter estimates can be found in S1 Appendix Table 5 and estimated cell means in Table 5.

Lookbacks from transitions at sentence boundaries were necessarily focused at a greater sentence distance than looking back from transitions before or within words. We found no effect of language. From our model of just transitions before and within words we found no evidence of a main effect of whether the transition was before or within words. However we did find some evidence of a main effect of language (BF = 4.76), with evidence that lookback is deeper into the text when writing in L2, both when transitions are within word (BF > 100) and before word (BF = 4.90).

## Discussion

The research that we have reported explored temporal patterns within the keystroke activity of adults’ spontaneous composition of multi-sentence text. Our study follows in a tradition of similar observational research [9,32,50–52]. We make three contributions. First previous studies have analyzed frequency and duration of IKIs that exceed a researcher-specified threshold (“pauses”). Our approach directly modeled duration and frequency of both long and short IKIs without the need for a priori threshold assumptions. Second, by capturing writers’ eye movements we have gone some way towards determining activity that occurs between keystrokes. Third, by comparing production in a language in which participants had and had not reached native-level competence we explored how the negative effects of production disfluency might affect tendency to lookback within existing text.

L2 texts were substantially shorter, less informationally dense, and less lexically diverse than texts written in the students first language. They were composed with fewer keystrokes, and substantially more of these keystrokes were associated with editing existing text.

Our focus, however, was not on these editing actions but on the processing associated with ongoing production of text. We have argued that in maximally fluent production processing upstream of finger movements provides information faster than it can be output as finger movements. Under these circumstances output is limited by the rate at which finger movements can be planned and executed. We found that for the experienced typists in our present study time to plan keystrokes had a mean of around 153 ms. In L1 53% of before-word IKIs were greater than would be expected just on the basis of time to move fingers. We interpret these as cases where the next word had not been fully planned in parallel with previous output. The additional planning overheads were relatively low, with mean duration of the slower transitions of just 433 ms. By contrast in L2 hesitation before words occurred in the vast majority of cases (88%) and recovery was more costly (estimated mean of 703 ms).

Lookback followed a similar pattern. In L1 writers looked back into their text (i.e., made two or more fixations behind the word that they had just typed) before 1 in 24 mid-sentence words. This increased to 1 in 9 words when they wrote in L2. Lookback when the participant was in the middle of writing a word was around three times more likely when they were writing in L2 (taking into account the fact that mean word length was greater in L2 than in L1). Lookback behavior, when it did occur, was similar in L1 and L2 in terms of both total lookback duration and number of words fixated, with participants taking a mean of around 1.5 s and fixating 4 or 5 words when looking back at the start of a word. Fewer words were fixated and fixation durations were shorter when looking back from mid-word positions. (Depth of lookback – mean distance in number of sentences from point of inscription of the most-fixated sentence during lookback – was greater in L2, but this is likely to be an artefact of participants producing shorter L2 sentences.)

Both hesitation and lookback were much more frequent before the start of sentences relative to before and within words. This was particularly the case in L2, with 94% of sentences preceded by hesitations which, again, were on average longer than in L1. Increased tendency to pause at sentence boundaries, relative to other locations within the text, is consistent with the findings of previous research in [7,8,45,46], as is a greater tendency for sentence-initial pausing in L2 relative to L1 [26,29]. Lookback was also much more common at sentence boundaries than at other locations with between 30% and 40% of sentences preceded by lookback in both L1 and L2. In this case we did find a language effect, with lookback sequences that had longer duration and involved fixating a larger number of words when participants wrote in L2.

These findings, taken together, are consistent with written composition resulting from processing that has the potential to run in parallel with output. The fact that in L1 47% of words and 22% of sentences were preceded by IKIs drawn from a distribution with an estimated mean of just 153 ms – a duration that we are interpreting as the time required to plan and execute finger movement – either means that these words were somehow produced without any advance planning or, more plausibly, that planning required for initiating the next word or sentence was completed in parallel with output of the preceding text. Orthographic, lexical and syntactic planning will all be less automatized in non-expert L2 writers, increasing the probability that retrieval will require central processing: the writer has to stop and explicitly search for the words needed to communicate their intended message. This has two consequences. Retrieval in most cases is not parallelized, resulting in a much greater tendency to hesitate before words. The processing that occurs during this hesitation – devoting explicit attention to word retrieval – is also likely to interfere with upstream processing associated with maintaining meaning. We interpret the much greater tendency to look back before words, in contexts where writers did not then make changes to their preceding text, as being associated in some and possibly most cases with reactivating memory traces and/or cueing retrieval of new content for what to communicate next. This account also is consistent with our finding that number of words fixated, when lookback occurred, was similar in L1 and L2: Assuming this is the function that lookback serves, then there is no particular reason why a larger number of words needs to be fixated in L2 than in L1 in order to cue what to say next.

A different pattern emerged at sentence boundaries with hesitation occurring in a substantial majority of cases in both languages and no difference in likelihood that these were associated with lookback. However, where lookback did occur this was more extensive in L2. One possible reason for this is that cueing new content – a semantic representation of what to say next – is going to be less easily achieved when the language that you are reading is relatively unfamiliar. It is perhaps also worth noting that although hesitation was common at sentence boundaries in both languages there remained 22% of cases in L1 where transition to the next sentence occurred with no hesitation at all (i.e., no longer than would be predicted by time required for finger movement; see also [10]). It is difficult to find an explanation for this other than in terms of planning what to write next in parallel with typing the previous sentence.

We argue, therefore, that our findings are consistent with the theory that lookback supports planning: Words read when writers look back into their text serve to cue what to say next, and that this function is particularly important as a way of reinstating content that is lost when writers struggle with lower-level features of their text.

Our findings may also be consistent, however, with lookback serving to monitor for errors. Although our analyses excluded transitions that ended with error correction this does not rule out the possibility that eye movement, we are observing is associated with error monitoring that fails to detect any errors. We do not have strong evidence against this argument, except to say that glancing back into the text very regularly, with fixations that hop about in the text, would seem a substantially less effective approach to detecting errors than less frequent but sustained reading over larger text spans.

### Pedagogical implications

Our focus in this study was on the fundamental cognitive processes that permit the production of multi-sentence spontaneous text. Our findings do not, we believe, have direct implications for L1 or L2 writing pedagogy. However, we recognize that some readers may be looking for clues in our findings as to how they or their students might write more effectively. We tentatively offer the following observations.

First, if, as we have argued, multi-sentence text production is a semi-parallel, just-in-time process, this suggests that writing instructors should be wary of interventions that might disrupt this fluent transfer of information down this cascade. For example, emphasizing the necessity of accurate spelling, or teaching explicit spelling rules, have the potential to introduce additional processing that intrudes on the smooth flow of information from message to finger movement. A writer who stops to remember the spelling rule “i before e except after c” might spell the word “deceive” correctly, but this may be at the cost of forgetting who was deceiving whom (i.e., at the cost of the writer’s intended meaning dropping out of their attentional focus). This is particularly likely to be an issue if the writer is already struggling with one or more processing components, as would be the case if writing in L2. The mechanism that we have described in this paper provides theoretical context to the “double challenge” that faces developing writers who must incorporate new instruction when already struggling to maintain fluent production [53].

Second, our findings point towards lookback into existing text serving to cue ongoing production: In situations where fluency is disrupted, which will inevitably occur even in competent L1 writers, glancing back into the text reinstates memory of what the writer was going to say next. These lookbacks are typically rapid, brief, and, we suspect, largely automatic. These features are likely to be necessary for lookback to support ongoing fluent production: the writer needs to rapidly refresh memory and then continue typing. There may be value, therefore, in instructors discouraging these lookbacks turning into more extended reading of existing text, at least during initial drafting. This finds some support in a study by Dux Speltz and Chukharev-Hudilainen [54]. Competent writers produced short essays under a fading text condition with text-already-written rapidly disappearing as the writer paused. This permitted brief lookback into existing text but prevented extended reading or error correction. Compared to controls, these students produced texts that were more error prone (something that could be fixed in a subsequent revision phase), but both were substantially longer and received higher holistic quality ratings.

Third, our findings point generally towards the potential value of writing instructors, or computer-assisted learning systems, knowing about students’ eye movements when composing text. The eye-tracking technology – both hardware and software – that we used in the present study, and described in more detail in [2], makes collecting these data in the classroom both feasible and reasonably affordable. Some preliminary studies suggest value in providing students with feedback on their lookback behavior both retrospectives, after completing their text [55,56] and concurrently during production [57].

### Conclusions

In conclusion, we believe that the research we report in this paper demonstrates two things. First, we have shown that methods for collecting and analyzing eye movement data that map gaze onto the emerging text, and methods for analysis of inter-keystroke intervals that go beyond counting pauses, provide valuable insight into the processes that underlie spontaneous written composition. Second, we have argued that a theory of the moment-by-moment processing that occurs during spontaneous composition based in semi-parallel, just-in-time processing is consistent with our findings.

Our present study did not, however, examine the association between the text that participants fixated during lookback and the semantic and linguistic features of what they immediately wrote. The combined eye-tracking and keystroke logging methods that we have described make this analysis possible, and we see this as an obvious target for future research. Examining semantic connections between lookback fixations and subsequent text production, however, requires a distinct analytical approach. While this is an area of ongoing work for us, it falls outside the scope of the present study. Future research should explore how semantic processing during lookback informs the production of new text and how this differs between L1 and L2 writing.

## Supporting information

S1 Appendix Mixture model formula and additional model statistics. (PDF)
