# Supplementary material for: Lookback supports semi-parallel, just-in-time processing in second language written composition
Source: PLoS One. 2025 Nov 3;20(11):e0334960. doi: 10.1371/journal.pone.0334960 (PMC12582431; doi:10.1371/journal.pone.0334960)
Supplement: S1 Appendix — (PDF) [file pone.0334960.s001.pdf]

S1 Appendix. Mixture model formula and additional model statistics.

To capture an understanding of interkey intervals as representing fluent or hesitant production, without specifying *a priori* pause thresholds, we modeled these intervals as follows:

$$\mathrm{IKI}_i \sim \theta_{\mathrm{language}[i],\mathrm{location}[i],\mathrm{participant}[i]}$$

$$\cdot\, \mathrm{LogN}\left(\beta + \delta_{\mathrm{language}[i],\mathrm{location}[i]} + u_{\mathrm{participant}[i]}, \sigma^2_{e'_{\mathrm{language}[i],\mathrm{location}[i]}}\right) +$$

$$(1 - \theta_{\mathrm{language}[i],\mathrm{location}[i],\mathrm{participant}[i]})$$

$$\cdot\, \mathrm{LogN}\left(\beta + u_{\mathrm{participant}[i]}, \sigma^2_{e_{\mathrm{language}[i],\mathrm{location}[i]}}\right)$$

where

$$u_{\mathrm{participant}} \sim N(0, \sigma^2_{\mathrm{participant}})$$

and constrained such that

$$\sigma^2_e, \sigma^2_{e'}, \sigma^2_{\mathrm{participant}} > 0$$

$$\sigma^2_{e'} > \sigma^2_e$$

$$0 < \theta < 1$$

$$\delta > 0$$

IKIs are modelled as belonging to one or other of two distributions. The second two lines of the formula represent a distribution for IKIs with shorter durations, modelled with just a random by-participant effect. The first two lines of the equation is a distribution for IKIs with longer (hesitant) durations, modelled with fixed effects for language and location in addition to the random by-participant effect. The additional $\delta_{\mathrm{language}[i],\mathrm{location}[i]}$ component represents the difference in

duration relative to shorter durations (δ is contrained to be greater than 1). θ is the esimated probability that the duration of a specific IKI was generated by the data-generating processes underlying hesitant IKIs – whether or not the IKI was a "pause". Across all IKIs within a language-by-location condition θ can be understood as the population (out-of-sample) estimate for the proportion of hesitant IKIs. Estimates of the means of the durations of fluent and hesitant IKIs are given, respectively, by β and by $\beta + \delta$.

Table 1

Parameter estimates (logit scale) for model predicting proportion of transitions associated with editing, by location and by language.

Predictor	Estimate with 95% PI	BF
Language (L1, L2)	-1.63 [-1.94 – -1.31]	> 100
Location 1 (before sentence vs. before word)	1.53 [1.31 – 1.75]	> 100
Location 2 (before word or sentence vs. within word)	3.65 [3.42 – 3.89]	> 100
Language : Location 1	0.03 [-0.19 – 0.25]	0.12
Language : Location 2	-0.41 [-0.64 – -0.18]	40.4

Table 2

Estimated effects from a finite mixture model predicting transition duration, with 95% PI.

Predictor	Duration (log_{10})	BF	Proportion (logit)	BF
Language (L1, L2)	-0.38 [-0.48, -0.26]	> 100	-1.49 [-1.89, -1.1]	> 100
Location 1 (before sentence vs. before word)	1.07 [0.99, 1.16]	> 100	0.93 [0.47, 1.41]	> 100
Location 2 (before word or sentence vs. within word)	1.1 [0.9, 1.25]	> 100	3.48 [3.03, 3.94]	> 100
Language : Location 1	0.15 [-0.02, 0.31]	0.39	0.51 [-0.43, 1.42]	0.86
Language : Location 2	0.09 [-0.18, 0.41]	0.16	0.63 [-0.23, 1.48]	1.22

Table 3

Parameter estimates for models predicting proportion of transitions that were associated with lookback and, where lookback occurred, number of words fixated. Estimate [95% PI], BF

Predictor	Probability of lookback (logit scale)	Number of words fixated ($\log_{10}$ scale)
Language (L1, L2)	-1.37 [-1.98 , -0.71], > 100	-0.32 [-0.63 , -0.01], 2.66
Location 1 (before sentence vs. before word)	4 [3.77 , 4.22], > 100	1.57 [1.43 , 1.71], > 100
Location 2 (before word or sentence vs. within word)	5.31 [5.15 , 5.49], > 100	3.81 [3.49 , 4.12], > 100
Language : Location 1	0.86 [0.64, 1.08], > 100	-0.22 [-0.36, -0.08], 21.1
Language : Location 2	0.02 [-0.15 , 0.19], 0.18	-0.26 [-0.57 , 0.06], 1.24

Table 4

Parameter estimates from a unimodal mixed effects model predicting lookback duration. Values are for estimated effect, 95% PI, and BF.

Predictor	Parameter estimate (log_{10} scale)	BF
Language (L1, L2)	-0.26 [-0.37, -0.16]	> 100
Location 1 (before sentence vs. before word)	-0.13 [-0.31, 0.05]	0.24
Location 2 (before word or sentence vs. within word)	0.04 [-0.09, 0.17]	0.08
Language : Location 1	0.58 [0.49, 0.67]	> 100
Language : Location 2	0.65 [0.58, 0.72]	> 100

Table 5

Parameter estimates for models predicting distance from point of inscription of most frequently fixated sentence during a lookback sequence. Estimate [95% PI], BF. Separate models for transitions occurring before-sentence and before- and within-words.

Predictor	Before sentences	Before and within words
Language (L1, L2)	-0.14 [-0.33, 0.06], 0.56	-1.01 [-1.46, -0.58] , > 100
Location (before word vs. within word)	-	0.11 [-0.16, 0.38] , 0.18
Language : Location	-	0.22 [-0.04, 0.49] , 0.51
